# Reproducibility crisis in isothermal amplification: lessons from benchmarking LAMP assays

**DOI:** 10.1128/spectrum.03235-25

**Published:** 2026-04-27

**Authors:** Lena Piglmann, Lena Campostrini, Regina Sommer, Alexander K. T. Kirschner, Rudolf Krska, Andreas H. Farnleitner, Claudia Kolm, Georg H. Reischer

**Affiliations:** 1 Institute of Chemical, Environmental and Bioscience Engineering, Working Area Molecular Diagnostics, IFA Tulln, TU Wien 210802 , Tulln, Austria; 2 ICC Interuniversity Cooperation Centre Water & Health, Vienna, Austria; 3 Centre for Pathophysiology, Infectiology and Immunology, Institute for Hygiene and Applied Immunology – Research Unit Water Microbiology, Medical University of Vienna 27271, Vienna, Austria; 4 Centre for Pathophysiology, Infectiology and Immunology, Institute for Hygiene and Applied Immunology – Research Unit Water Hygiene, Medical University of Vienna 27271, Vienna, Austria; 5 Division Water Quality and Health, Karl Landsteiner University of Health Sciences 646493, Krems, Austria; 6 Institute of Bioanalytics and Agro-Metabolomics, Department of Agricultural Sciences, IFA Tulln, University of Natural Resources and Life Sciences Vienna (BOKU), Tulln, Austria; 7 Institute for Global Food Security, School of Biological Sciences, Queens University Belfast, Belfast, Northern Ireland, United Kingdom; 8 Institute of Chemical, Environmental and Bioscience Engineering, Research Group Microbiology and Molecular Diagnostics 166/5/3, TU Wien, Vienna, Austria; Connecticut Agricultural Experiment Station, New Haven, Connecticut, USA

**Keywords:** isothermal amplification, LAMP, reproducibility, assay characteristics, molecular diagnostics, *Pseudomonas aeruginosa*

## Abstract

The number of published isothermal amplification assays has increased substantially in recent years. Unfortunately, no harmonized guidelines, such as the Minimum Information for Publication of Quantitative Real-Time PCR Experiments guidelines, exist for publishing these methods, often resulting in incomplete reporting of assay composition and performance. In this study, we systematically evaluated nine published loop-mediated isothermal amplification (LAMP) assays for the detection of *Pseudomonas aeruginosa*. We aimed to assess whether publications provide (i) sufficient information on assay composition to allow implementation and reproduction and (ii) robust data on assay performance to evaluate their applicability. Assays were screened for basic functionality, analytical specificity, sensitivity, and limit of detection (LOD) in head-to-head experiments with qPCR. Four assays lacked essential composition details, and almost all did not report DNA concentrations or replicate numbers. Only six assays consistently amplified target DNA. Analytical specificity testing with 19 non-target strains contradicted previously reported 100% specificity, with only 3 maintaining specificity above 90% in our evaluation. Sensitivity testing with 13 *P. aeruginosa* strains confirmed 100% sensitivity for two assays. However, LOD experiments revealed significantly higher values than originally reported, with qPCR outperforming all LAMP assays. These findings highlight substantial discrepancies between published data and real-world assay performance. The absence of standardized formats, consistent units, and complete methodological details undermines replicability. Although this study focused on *P. aeruginosa*, the identified issues are widely relevant across different microbial targets. We advocate for increased publication standards and quality controls to ensure transparency, utility, and comparability of isothermal amplification assays and to support their translation into clinical and environmental applications.

**IMPORTANCE:**

Over the past decade, numerous isothermal amplification-based assays for the detection of pathogens or health-relevant microorganisms have been proposed, each claiming progress from the state-of-the-art and applicability to both clinical specimens and/or environmental samples. However, many published assays lack essential methodological details, and reported performance metrics are often inconsistent or incomparable. Using *Pseudomonas aeruginosa* as a representative model organism, we set out to test whether all details required for implementing assays were provided by original publications and if important assay characteristics were reproducible. The results of this systemic benchmarking of nine published loop-mediated isothermal amplification assays revealed major discrepancies between reported and experimentally measured performance in terms of specificity, sensitivity, and limit of detection. By highlighting the crucial elements that need to be reported, this work aims to improve the transparency, reproducibility, and overall quality of isothermal amplification assays, fostering their broader application across different research settings.

## INTRODUCTION

The detection of microbial pathogens has traditionally been based on the widely established method of microbiological cultivation (1). Cultivation-based diagnostics are easy to use, inexpensive, as well as highly standardized, but they can take several days until results are available and confirmed (2, 3). In contrast, molecular techniques can offer faster, more sensitive, and often more specific detection (4, 5). PCR, especially in its quantitative format (qPCR), is widely considered as the gold standard for nucleic acid detection (6). However, applying PCR requires a molecular biology laboratory with expensive thermal cyclers and trained personnel (7), which severely limits point-of-care applications as well as on-site testing in remote or low-resource settings by untrained personnel. To overcome this drawback, isothermal amplification methods have emerged over the past decades. These techniques can amplify DNA with high specificity at a constant temperature, eliminating the need for complex thermal cyclers for heating and cooling steps and shortening the time to result (8). Therefore, they can be applied in resource-limited settings and are not dependent on highly trained personnel (9). The loop-mediated isothermal amplification (LAMP) technique developed in the year 2000 by the group of Notomi (10) is nowadays the most widely applied method. It is based on a DNA polymerase with strand-displacement activity and four to six primers, which bind to six to eight locations of the target DNA, ensuring a high level of sequence selectivity (11). Gel electrophoresis or the addition of DNA-intercalating fluorescent dyes can be applied for the detection of LAMP products (12). LAMP has a high degree of robustness due to the use of *Bacillus stearothermophilus* DNA polymerase (Bst), which is less susceptible to inhibitory substances than other polymerases, promoting application on difficult samples such as untreated urine or complex matrices found in food, feed, or polluted water samples (13, 14).

These applications require a high level of specificity and sensitivity to detect the target nucleic acid in a complex background of sample matrix and other organisms. Particular attention must be paid to the detection limit, as target organisms are often only present in very low concentrations but nevertheless have a strong impact on public health. These assay characteristics comprising analytical specificity, sensitivity, and limit of detection (LOD) are critical pieces of information needed to decide if an assay is suitable for the desired application in an environmental or clinical context. Analytical specificity is defined as the capability to differentiate the target organism from non-target organisms (15). Assay sensitivity is the ability of an assay to detect the specific DNA sequence in samples containing the target organism (16). The LOD is characterized by the lowest quantity of an analyte in a sample that can be reproducibly detected (17).

In addition to data on assay performance characteristics, complete and transparent information on reaction composition, primer/probe sequences, sample concentrations, as well as the number of replicates and controls is essential for reliable implementation of a molecular diagnostic assay (18). To promote reproducibility and provide a consistent framework for assay development and evaluation, the Minimum Information for Publication of Quantitative Real-Time PCR Experiments (MIQE) guideline was published in 2009 (19). This guideline defines the minimum set of information required to evaluate a qPCR assay, covering aspects from description of sample material, experimental design, protocols, and instruments to data analysis (20). Adherence to such standards facilitates independent assessment, replication, and application of published assays. However, no comparable guideline currently exists for publishing isothermal amplification methods and comprehensive reporting of assay information, and performance is often neglected in the field. Over the past decade, numerous isothermal amplification-based assays for the detection of bacterial indicators and pathogens have been proposed, often claiming superior sensitivity, specificity, limit of detection, and broad applicability across various sample types. Yet, in contrast to qPCR methods, only a small fraction of these assays have been independently validated or applied in studies addressing real-world clinical or environmental challenges.

To explore the gap between reported potential and practical implementations, we selected *Pseudomonas aeruginosa* as a model organism. *P. aeruginosa* is an aquatic and soil bacterium found ubiquitously in the environment and has been the subject of numerous published LAMP assays. This bacterium is an opportunistic pathogen in humans, and one of its most serious characteristics is its ability to develop multidrug-resistant strains (21). Its ability to form biofilms, e.g., in sink and shower drains, is particularly problematic as it ensures long-term persistence (22, 23). Its ubiquity, genetic diversity, and resistance traits make it a suitable representative case to evaluate general issues in isothermal assay development and reporting. The aim of this study was to assess whether publications proposing novel isothermal amplification assays meet the requirements of providing all the (i) information on assay composition that is necessary to implement and reproduce an assay, as well as the (ii) information on assay performance that is required to judge an assay’s usefulness for a specific application. We are benchmarking these assays against a qPCR reference protocol, which in this context means comparability in terms of specificity, sensitivity, and limit of detection (not in terms of being quantitative). By applying consistent evaluation criteria, we aim to reveal common gaps in assay reporting and to propose a structured approach that may serve as a practical guideline for future isothermal diagnostic assay development.

## RESULTS

### Assay information and reported performance

The detailed literature search for published loop-mediated isothermal amplification assays for the detection of *P. aeruginosa* resulted in 12 different LAMP assays. Three assays had to be excluded as the authors did not publish primer sequences and/or reaction compositions. The remaining nine assays were included in this study. All available information regarding assay composition and conditions, tested samples, specificity, sensitivity, and LOD was collected, as listed in Table 1. All nine publications reported their LAMP product detection method and the sample types used for assay evaluation. Detection methods included lateral flow strips, gel electrophoresis, chromogenic agents, fluorescent dyes, as well as real-time fluorescence measurements. In most of the assays, purified DNA served as input material for assay evaluation. In some cases, DNA extracts from water, soil, as well as from clinical samples such as urine were used in specificity and/or sensitivity experiments. Information on specificity and sensitivity was provided by eight out of nine publications. All publications carried out limit of detection experiments. Most publications did not specify the DNA concentrations (e.g., total genomic DNA mass, genome, or target gene copies) in target and non-target samples used for testing (eight out of nine). In many cases, the number of replicate reactions was also omitted (four out of nine for LOD experiments, six out of nine for specificity and sensitivity evaluations). All but three publications reported values of 100% for specificity and sensitivity. The number of samples used for these assessments varied between 8 and 380. Reported LODs are difficult to compare since they varied widely in units, including CFU/mL, CFU/reaction, bacteria/reaction, weight of DNA/reaction, and copies/reaction.

**TABLE 1:** Summary of assay characteristics of the different LAMP assays targeting *P. aeruginosa*^*a*^*^,^*^*b*^

Title	Author	Year of publication	Detection method	Tested samples	Specificity	Sensitivity	LOD	Cited and used by
Point-of-care and visual detection of *P. aeruginosa* and its toxin genes by multiple LAMP and lateral flow nucleic acid biosensor	Chen et al. (24)	2016	Lateral flow nucleic acid biosensor, gel electrophoresis	Purified DNA, water, and soil samples	N/A	N/A	20 CFU/mL	Cited by 110 publications, primers used by none
Rapid and sensitive detection of *Pseudomonas aeruginosa* in bottled water by loop-mediated isothermal amplification	Zhang et al. (25)	2012	Chromogenic reagent	Purified DNA, bottled water spiked and non-spiked	100% (27)	100% (81)	15.7 CFU/mL pure culture, 3.1 CFU/250 mL spiked water	Cited by 15 publications, primers used by 1
Development and application of a loop-mediated isothermal amplification method on rapid detection of *Pseudomonas aeruginosa* strains	Zhao et al. (26)	2011	SYBR Green (visual and UV detection), gel electrophoresis	Purified DNA, clinical samples	100% (174)	97.6% (252)	100 fg DNA/tube, 10 CFU/reaction	Cited by 81 publications, primers used by 1
Development of uracil-DNA-glycosylase-supplemented loop-mediated isothermal amplification coupled with nanogold probe (UDG-LAMP-AuNP) for specific detection of *Pseudomonas aeruginosa*	Manajit et al. (27)	2018	Nanogold probe, gel electrophoresis	Purified DNA, spiked contact lenses	100% (22)	100% (16)	3 CFU/reaction for purified DNA, 2 CFU/reaction for spiked contact lenses	Cited by 15 publications, primers used by none
Establishment of loop-mediated isothermal amplification for rapid detection of *Pseudomonas aeruginosa*	Li et al. (28)	2018	SYBR Green, gel electrophoresis	Purified DNA, mouse blood infected with *P. aeruginosa*	100% (170)	100% (150)	One bacterium/reaction	Cited by 17 publications, primers used by none
Rapid detection of *Pseudomonas aeruginosa* in mouse feces by colorimetric loop-mediated isothermal amplification	Goto et al. (29)	2009	Colorimetric	Purified DNA, clinical samples, spiked mouse feces	100% (40)	100% (49)	12 CFU/reaction for gDNA, 3.25 CFU/reaction for feces	Cited by 57 publications, primers used by 2
A loop-mediated isothermal amplification with a nanoparticle-based lateral flow biosensor assay to detect *Pseudomonas aeruginosa* in endophthalmitis	Dong et al. (30)	2021	Lateral flow biosensor, colorimetric	Purified DNA, clinical samples	100% (21)	100% (15)	100 fg/test (~14 copies)	Cited by five publications, primers used by none
A LAMP-based system for rapid detection of eight common pathogens causing lower respiratory tract infections	Si et al. (31)	2021	Fluorescence measurement	Purified DNA, sputum samples	78.68% (380)	92.57% (148)	10^3^–10^4^ CFU/mL	Cited by seven publications, primers used by one
Uracil-DNA-glycosylase-assisted loop-mediated isothermal amplification for detection of bacteria from urine samples with reduced contamination	Zeng et al. (32)	2020	Fluorescence measurement, colorimetric	Purified DNA, urine samples	Without UDG: 88.1% (42), with UDG: 94.8% (58)	Without UDG: 100% (8), with UDG: 100% (12)	10^4^ CFU/mL	Cited by 13 publications, primers used by none

^*a*^ The indication N/A means that no information was available for this section.

^*b*^ Displayed are the detection method, tested samples, and specificity as well as sensitivity (here numbers of used non-target or target strains are shown in brackets), limit of detection, and if assays have been cited and/or used by other users.

The nine selected assays targeted different genes. The *ecfX* and *oprL* genes were targeted by three assays each, while *oprI*, exotoxin A, as well as a hypothetical protein gene were each targeted by one assay. The employed primer sequences are summarized in Table 2. The reaction compositions of all assays were extracted and are summarized (Table S1). Six out of the nine protocols provided all necessary information to fully reproduce the assay. In the case of three assays, buffer components and their concentrations were incompletely reported, and additionally, two studies omitted primer concentrations. Most assays used uniform units of Bst 2.0 DNA Polymerase Large Fragment and Isothermal Amplification Buffer (both New England Biolabs, Ipswich, USA). Common additives included betaine, dNTPs, and MgSO_4_.

**TABLE 2:** Oligonucleotides as well as temp.^*d*^ and time used for the qPCR and LAMP reactions targeting different genes of *P. aeruginosa*

Assay and reference	Oligonucleotide	Sequence 5′−3′	Temp. and time
16S_qPCR (33)	8F	AGAGTTTGATCCTGGCTCAG	
	338R	TGCTGCCTCCCGTAGGAGT	
GyrB_qPCR (34)	pa722_F	GGCGTGGGTGTGGAAGTC	
	pa899_R	TGGTGGCGATCTTGAACTTCTT	
	pa746_MGB	FAM-TGCAGTGGAACGACA-NFQ-MGB	
Chen et al. (24)^*a*^	ecfX-F3	GGATGAGCGCTTCCGTG	65°C for 40 min
	ecfX-B3	AAGTTGCGGGCGATCTG	
	ecfX-FIP	CTTGCGCAGAAGCGCAGCGTTCCGTCTCGCATGCCTA	
	ecfX-BIP	GCCGACCTCGCCCAGGATAGCTCGACCGATTGCCG	
Zhang et al. (25)	ecfX-F3	GGATGAGCGCTTCCGTG	65°C for 60 min
	ecfX-B3	AAGTTGCGGGCGATCTG	
	ecfX-FIP	CTTGCGCAGGAAGCGCAGC-GTTCCGTCTCGCATGCCTA	
	ecfX-BIP	GCCGACCTCGCCCAGGATA-GCTCGACCGATTGCCG	
Zhao et al. (26)	oprI-F3	CTGGCTGCTGTTCTGG	65°C for 45 min
	oprI-B3	CGCTCGTTAGCCTCGT	
	oprI-FIP	CTGCGTCTTCGGTAGCGG-GGTTGCAGCAGCCACT	
	oprI-BIP	TCAGGCTCGCGCTGACGA-AGTCTGCTGAGCTTTCTGAG	
	oprI-LF	TCTTTGGCTTCGAGCAGACT	
	oprI-LB	GCCTATCGCAAGGCTGACGAA	
Manajit et al. (27)	ecfX-F3	TCCGTGGTTCCGTCTCG	65°C for 60 min
	ecfX-B3	AAGTTGCGGGCGATCTG	
	ecfX-FIP	TGCCCAGGTGCTTGCGCATTTTCATGCCTATCAGGCGTTCC	
	ecfX-BIP	GCCGACCTCGCCCAGGATATTTTGCTCGACCGATTGCCG	
Li et al. (28)	Hypothetic_Protein-F3	CAAGCGCAAGATAGTCGCC	65°C for 30 min
	Hypothetic_Protein-B3	TCCGCTTGAACAGGCTGGTG	
	Hypothetic_Protein-FIP	GAAGATATCCGGCTGGTTGCTTTTCAAGAGGGAATGCCGCAGT	
	Hypothetic_Protein-BIP	AACGGATCATCGGCATCCTGGTTTTCATCGCCGTCCACAGGTAGA	
Goto et al. (29)	oprL-F3	GCGTTGCCGCCAACAATG	63°C for 60 min
	oprL-B3	CATGCGGGCAACCTCTC	
	oprL-FIP	GTTGTCACCCCACCTCCGGGCGGCAACGTTCCTCC	
	oprL-BIP	CTCCGTGCAGGGCGAACTGCAGGCGAGCCAACTC	
	oprL-LF	ACCTGCCGTGCCATACC	
	oprL-LB	GTTCATGCAGCTCCAGCAG	
Dong et al. (30)^*b*^	oprL-F3	AGCCGGAAGCCATGC	65°C for 60 min
	oprL-B3	AACGCCCTGCAGCACC	
	oprL-FIP	TGGCCTTCCAGCACTACGCGTCTGGACGTACACGCGAAAG	
	oprL-BIP	ACCGACGAACGCGGCATAGCGCTGAACGGCCTTG	
	oprL-LF	TGACCGCTGCCTTTCA	
	oprL-LB	CGAGTACAACATGGCTCT	
Si et al. (31)	Exotoxin_A-F3	GCGCGATGCCACCTTCT	63°C for 45 min
	Exotoxin_A-B3	TGCAGCGTTGCTGGGC	
	Exotoxin_A-FIP	CCATGACCACGCTGACCCCGTTTTCACGAGAGCAACGAGATGC	
	Exotoxin_A-BIP	CGGGAAAAGCGCTGGAGCGAATTTTGAGGTAGTTGTAGACCCCGT	
	Exotoxin_A-LB	GCAAGGTGTTGTGCCTGCTC	
Zeng et al. (32)^*c*^	oprL-F3	GCGTTGCCGCCAACAATG	63°C for 60 min
	oprL-B3	CATGCGGGCAACCTCTC	
	oprL-FIP	GTTGTCACCCCACCTCCGGGCGGCAACGTTCCTCC	
	oprL-BIP	CTCCGTGCAGGGCGAACTGCAGGCGAGCCAACTC	
	oprL-LF	ACCTGCCGTGCCATACC	
	oprL-LB	GTTCATGCAGCTCCAGCAG	

^*a*^ Originally, FIP contained modified biotin at 5′, and BIP contained modified FITC on 5′.

^*b*^ Originally, FIP was labeled with biotin and LF with FITC.

^*c*^ Primers originally from Goto et al., (29) but as the reaction composition was different, we decided to include them anyway.

^*d*^ Temp., temperature.

### Assay performance: basic functionality of assays

As the first step, a comparative assessment of all nine LAMP assays via fluorescence monitoring was conducted. To this end, each assay was tested with a 1:10 dilution series of genomic DNA from *P. aeruginosa*-type strain ATCC 10145, ranging from 10^6^ to 10^2^ genomic copies per reaction (gc/rxn), analyzed in triplicate. All reactions were carried out in a total reaction volume of 25 µL, including 0.5 µL LAMP dye (New England Biolabs) and 2.5 µL sample DNA. Wherever possible, the original published reaction conditions were followed. If important information was missing, the standard conditions described in Material and Methods were used. As a reference method, the qPCR assay of Lee et al. (34) was chosen and also tested with the dilution series, resulting in the successful amplification of all samples.

Overall, six out of nine assays successfully amplified at least one dilution of the DNA. The assay published by Zeng et al. (32) yielded amplification in the first test run but not in the second. In addition, the no-template controls for this assay were positive on multiple occasions. Although the results were inconclusive, the assay was retained for further analysis. In contrast, the assays by Chen et al. (24) and Zhang et al. (25) did not detect *P. aeruginosa* DNA in any dilution and were therefore excluded from further testing.

### Assay performance: assessment of analytical specificity and assay sensitivity

Two of the most important criteria in establishing a molecular biological detection method are analytical specificity and sensitivity, especially in the context of clinical and environmental samples. To assess these characteristics for the selected LAMP assays and a qPCR reference method, we tested genomic DNA from target (*n* = 12) and non-target (*n* = 19) strains. The resulting sensitivity and specificity values were calculated using the published mathematical terms by Lemmon and Gardner (35). Additionally, the qPCR reference assay was also tested for its specificity and sensitivity, which were both determined as 100%.

#### Assessment of analytical specificity

Of the seven LAMP assays tested in this phase, five were originally reported to have 100% specificity. Si et al. (31) published a value of 78.68%; Zeng et al. (32) stated that the addition of UDG increased the specificity from 88.10% to 94.80% (Table 1). However, our experimental evaluation revealed that all seven assays gave at least one false-positive result for a non-target strain (Table 3). Only three assays reached an experimentally confirmed specificity above 90% and were retained for subsequent sensitivity evaluation.

**TABLE 3:** Results of the analytical specificity tests performed with the seven different LAMP assays^*a*^*^,^*^*b*^

	Zhao et al. (26)	Manajit et al. (27)	Li et al. (28)	Goto et al. (29)	Dong et al. (30)	Si et al. (31)	Zeng et al. (32)
*Escherichia coli*	**1/3**	0/3	**1/3**	0/3	0/3	**1/3**	0/3
*Enterococcus faecalis*	0/3	0/3	**2/3**	0/3	0/3	0/3	**1/3**
*Enterococcus faecium*	0/3	0/3	0/3	0/3	0/3	0/3	**2/3**
*Legionella anisa*	0/3	0/3	**1/3**	0/3	0/3	0/3	**3/3**
*Staphylococcus saprophyticus*	0/3	0/3	**2/3**	0/3	0/3	0/3	**3/3**
*Klebsiella pneumoniae*	**1/3**	0/3	0/3	0/3	0/3	0/3	**2/3**
*Proteus mirabilis*	0/3	0/3	0/3	0/3	0/3	0/3	**2/3**
*Pseudomonas putida*	0/3	0/3	0/3	**1/3**	0/3	0/3	**1/3**
*Pseudomonas fluorescens*	0/3	0/3	**1/3**	0/3	0/3	0/3	**3/3**
*Staphylococcus aureus*	**1/3**	0/3	**1/3**	0/3	0/3	0/3	**1/3**
*Streptococcus bovis*	0/3	0/3	**3/3**	0/3	0/3	0/3	**2/3**
*Citrobacter freundii*	0/3	0/3	0/3	0/3	0/3	0/3	**2/3**
*Acinetobacter baumannii*	0/3	0/3	**1/3**	0/3	0/3	0/3	**2/3**
*Vibrio cholerae*	0/3	0/3	0/3	0/3	**1/3**	0/3	**3/3**
*Vibrio mimicus*	**1/3**	0/3	0/3	0/3	0/3	0/3	**3/3**
*Vibrio parahaemolyticus*	**1/3**	0/3	0/3	0/3	0/3	0/3	**2/3**
*Vibrio vulnificus*	**1/3**	0/3	0/3	0/3	0/3	0/3	**3/3**
*Vibrio fluvialis*	0/3	0/3	**2/3**	0/3	0/3	0/3	**1/3**
*Vibrio alingolyticus*	0/3	**1/3**	0/3	**1/3**	0/3	0/3	**2/3**
Specificity (%)	68.43	94.74	47.37	89.47	94.74	94.74	5.26

^*a*^ The result for each non-target strain was considered false positive if at least one out of the three replicates was false positive (shown in bold). In the last line of the table, the overall calculated specificity is provided as percentages.

^*b*^ Each non-target strain was assessed with a genomic DNA concentration of 10^3^ genome copies/reaction and in triplicates.

#### Assessment of assay sensitivity

The sensitivity of the three remaining assays was tested on various environmental and clinical strains of *P. aeruginosa*. This pathogen is known for its high genomic variability, leading to the decision to assess the sensitivity of the LAMP assays and the qPCR reference method with a wide range of different isolates.

Both Manajit et al. (27) and Dong et al. (30) reported 100% sensitivity, based on testing 16 and 14 target strains, respectively (Table 1). In the publication of Si et al. (31), a value of 92.57% was stated (Table 1). Consistent with the original published sensitivity values of the assays by Manajit et al. (27) and Dong et al. (30), our experiments confirmed 100% sensitivity across all tested *P. aeruginosa* strains (Table 4). The assay of Si et al. (31) was not able to detect environmental isolates 4 and 6; therefore, it was excluded from further limit of detection experiments.

**TABLE 4:** Results of the assay sensitivity tests performed with the three different LAMP assays^*a*^*^,^*^*b*^

	Manajit et al. (27)	Dong et al. (30)	Si et al. (31)
*P. aeruginosa* type strain ATCC 10145	3/3	3/3	3/3
*P. aeruginosa* environmental isolate 1	3/3	3/3	3/3
*P. aeruginosa* environmental isolate 2	3/3	3/3	3/3
*P. aeruginosa* environmental isolate 3	3/3	3/3	3/3
*P. aeruginosa* environmental isolate 4	3/3	3/3	**0/3**
*P. aeruginosa* environmental isolate 5	3/3	3/3	3/3
*P. aeruginosa* Environmental Isolate 6	3/3	3/3	**0/3**
*P. aeruginosa* clinical isolate 1	3/3	3/3	3/3
*P. aeruginosa* clinical isolate 2	3/3	3/3	3/3
*P. aeruginosa* clinical isolate 3	3/3	3/3	3/3
*P. aeruginosa* clinical isolate 4	3/3	3/3	3/3
*P. aeruginosa* clinical isolate 5	3/3	3/3	3/3
*P. aeruginosa* clinical isolate 6	3/3	3/3	3/3
Sensitivity (%)	100	100	84.62

^*a*^ The result for each target strain was considered false negative if at least one out of the three replicates was false negative (shown in bold). In the last line of the table, the overall calculated sensitivity is provided as percentages.

^*b*^ Each target strain was assessed with a genomic DNA concentration of 10^3^ genome copies/reaction and in triplicates.

### Assay performance: assessment of the analytical limit of detection (LOD_95_)

The analytical limit of detection is of high importance for molecular diagnostics, as samples often only contain very low concentrations of the target organism. In the publications by Manajit et al. (27) and Dong et al. (30), detection limits were determined using a similar approach. Both used 10-fold serial dilutions of *P. aeruginosa* genomic DNA and defined the detection limit as the lowest concentration yielding positive results. For the assay of Manajit et al. (27), an LOD of 1.6 × 10^3^ CFU/reaction or equivalent to 3 CFU/reaction was stated; the assay of Dong et al. (30) declared a limit of detection of 100 fg or equivalent to 14 copies (Table 1). However, Dong et al. (30) did not specify the *P. aeruginosa* ATCC strain used in the assay but did measure the samples in three replicates. Manajit et al. (27) specified the *P. aeruginosa* strain (PA07), but no information on measured replicates was provided.

For our limit of detection experiments, we tested the two remaining LAMP assays and the qPCR reference method using isolated genomic DNA from three *P. aeruginosa* strains: the type strain ATCC 10145, clinical isolate 1, and environmental isolate 1. The concentration of these three different genomic DNA samples was quantified directly after DNA extraction with a qPCR assay targeting the V1–V2 region of the 16S rRNA gene with primer binding sites universal to all bacteria (33). Subsequently, twofold serial dilutions were prepared for all three *P. aeruginosa* strains. The respective concentrations were each measured in 12 replicate reactions. A logistic regression model was used for the statistical determination of the threshold where 95% of the replicates delivered a positive signal (LOD_95_) (7, 9). The modeled LOD_95_ values of the qPCR reference method were 13, 39, and 39 gc/rxn for ATCC 10145, environmental isolate 1, and clinical isolate 1, respectively (Fig. S1a through c).

**Fig 1:**
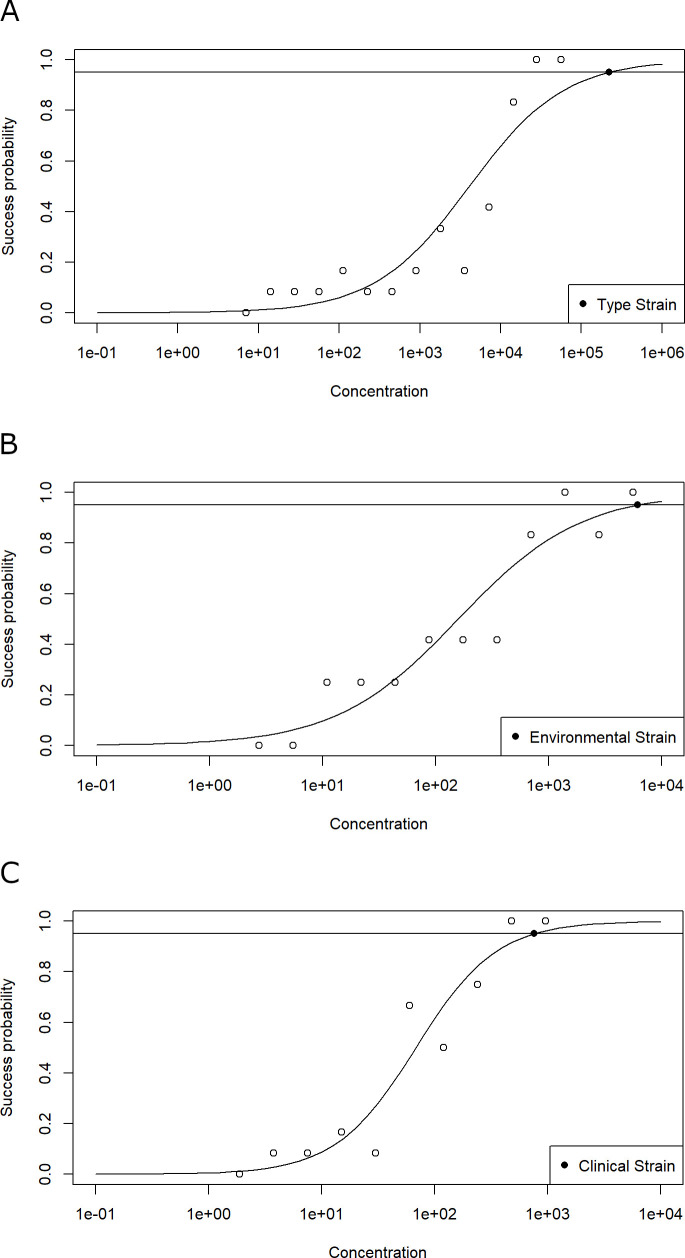
(**A–C**) Graphs showing the results for the LAMP assay from Manajit et al. (27), obtained from the analysis of the *P. aeruginosa* gDNA dilution series. The horizontal line with the filled symbol indicates the limit of detection where 95% of the replicates delivered a positive signal (LOD_95_). For this statistical determination, a logistic regression model was used. The modeled LOD_95_ of the LAMP assay detecting ATCC 10145, environmental isolate 1, and clinical isolate 1 was 2.21 × 10^5^, 6.17 × 10^3^, and 7.62 × 10^2^ gc/rxn, respectively.

**Fig 2:**
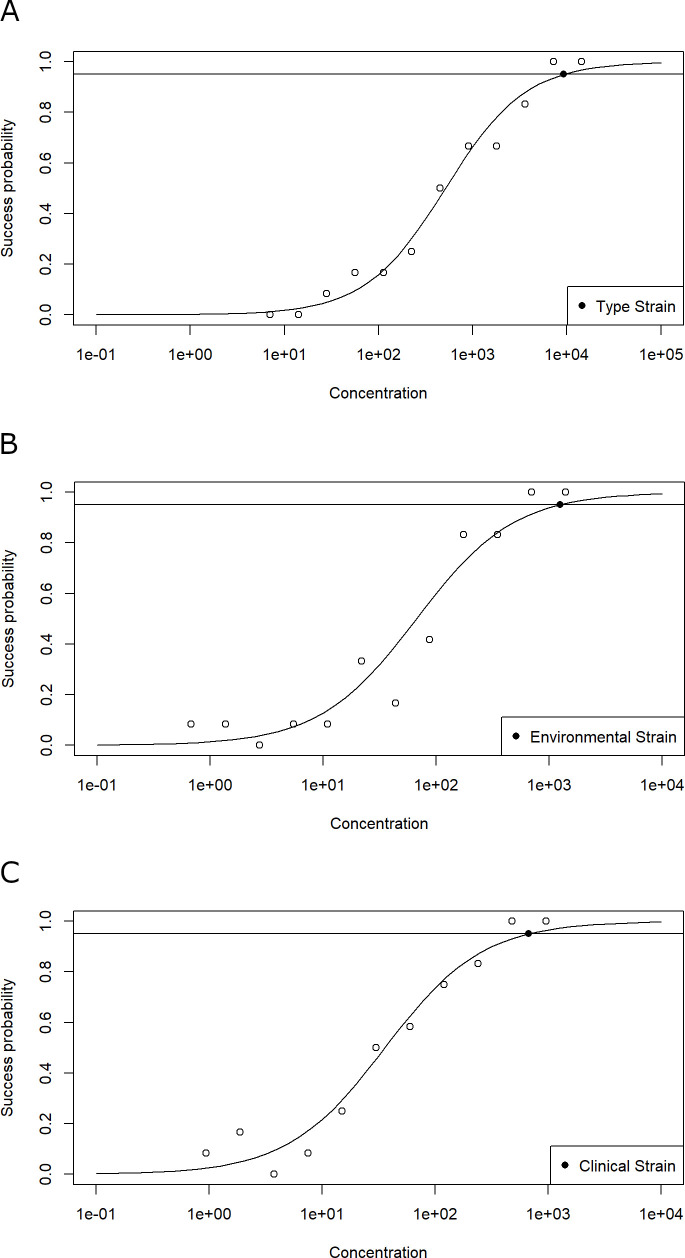
(**A–C**) Graph showing the results for the LAMP assay from Dong et al. (30), obtained from the analysis of the *P. aeruginosa* gDNA dilution series. The horizontal line with the filled symbol indicates the limit of detection where 95% of the replicates delivered a positive signal (LOD_95_). For this statistical determination, a logistic regression model was used. The modeled LOD_95_ of the LAMP assay detecting ATCC 10145, environmental isolate 1, and clinical isolate 1 was 9.25 × 10^3^, 1.25 × 10^3^, and 6.75 × 10^2^ gc/rxn, respectively.

For the LAMP protocol of Manajit et al. (27), the limits of detection were calculated and resulted in 2.21 × 10^5^ gc/rxn for the *P. aeruginosa* type strain ATCC 10145, 6.17 × 10^3^ gc/rxn for the environmental isolate 1, and 7.62 × 10^2^ gc/rxn for clinical isolate 1 (Fig. 1A through C). A similar trend was observed for the assay of Dong et al. (30), which showed the highest LOD_95_ for *P. aeruginosa* type strain ATCC 10145 with 9.25 × 10^3^ gc/rxn, followed by environmental isolate 1 with 1.25 × 10^3^ gc/rxn, and clinical isolate 1 with 6.75 × 10^2^ gc/rxn (Fig. 2A through C). In direct comparison, both LAMP assays exhibited substantially higher LOD_95_ values than the qPCR reference method. This difference is most evident in the type strain ATCC 10145, where the qPCR assay reached an LOD_95_ of just 13 gc/rxn, compared to 2 × 10^5^ and 9 × 10^3^ gc/rxn for the assays by Manajit et al. (27) and Dong et al. (30), respectively.

## DISCUSSION

Our systematic evaluation revealed widespread deficiencies in the reporting of LAMP assay protocols. Four out of the nine published assays lacked sufficient methodological detail to allow accurate reproduction by other users. In particular, details regarding the buffer components as well as primer concentrations were frequently missing. Almost none of the publications mentioned the DNA concentrations used for testing target and non-target samples, even though it is essential to ensure valid interpretation. In many cases, the number of replicate reactions used in specificity, sensitivity, and limit of detection experiments was also missing. These details are particularly important in environmental applications, where a wide variety of background organisms can be expected, and the concentration of the target organism can be low. The reporting gaps translated into unreliable assay performance and lack of reproducibility. Only six out of nine assays reliably amplified target genomic DNA during initial screening. Analytical specificity deviated remarkably from published results. Although five out of seven tested assays originally claimed 100% specificity (Table 1), in our experiments, they all yielded at least one false-positive result for a non-target strain. Only three assays achieved specificity values above 90% (Table 3), despite a considerable overlap in strain selection with the original publications (26, 28, 30–32). In many cases, information on strains used for testing was absent, even though verifiable specification of tested strains is essential for the interpretation of published values and for assessing assay performance and relevance. Sensitivity testing of the three most specific assays revealed 100% sensitivity for two of the assays (Table 4), consistent with published results (27, 30). For the third assay, a sensitivity value of 92.57% was reported, being higher than our result of 84.62% sensitivity (31). For LOD_95_ determinations, we employed defined genome copy numbers, twofold dilution steps, 12 technical replicates per dilution, and logistic regression to calculate detection probabilities. In contrast, the original publications used 10-fold serial dilutions and undefined strain sources, and one did not report replicate numbers. Additionally, no details were given on variability in measurements and reproducibility. The use of a statistical model and the declaration of the required detection probability are essential for the correct interpretation and reproducibility of the data (36). Without application of statistical models considering stochastic distribution of true positives at low target gene copy numbers, LOD determination is not informative, even if replicate numbers are given (37). In particular, at concentrations below 100 copies per reaction, variability between the technical replicates will occur, increasing from 10% to 200% coefficient of variation when approaching the LOD (38, 39). Measured LOD_95_ values for both LAMP assays were substantially higher than reported, particularly for the type strain ATCC 10145 (Fig. 1A and 2A). There are also considerable differences in LODs for different isolates, reemphasizing the importance of defining the strains used in testing. Units of measurement regarding the LOD also varied widely, complicating comparisons (e.g., CFU/reaction, DNA mass, and genome copies) (Table 1).

The need for defined standard protocols and harmonized units is mirrored by other studies comparing isothermal amplification assays. Wang et al. (40) compared 12 LAMP assays for the detection of pathogenic bacteria and stated that all 12 assays were unsuitable, as they observed false-positive results for all of them (40). Similar problems were encountered in another comparison study with LAMP assays for the detection of seven main serogroups of *Escherichia coli* (41). All assays showed non-specific amplification in the sensitivity evaluation, and the specificity was thereby not assessed. In the study by Desai et al., two isothermal amplification-based SARS-CoV-2 tests, including an FDA-approved assay, were evaluated for their clinical performance and operational characteristics. They found low sensitivity, variation in the performance of the two assays across diverse study sites, and notable differences compared with other studies assessing those assays (42).

Isothermal amplification methods have come into wide use over the course of the last two decades, and many different protocols are available, all with their own advantages and limitations. For environmental and clinical applications, it is particularly important that DNA amplification assays are specific, sensitive, and have a limit of detection suitable for the desired application. As a basis for determining these necessary assay performance characteristics, all details required for a reliable reproduction of a molecular diagnostic assay need to be provided. However, as is evident in this study and across the whole scientific field, there is currently a lack of good scientific practice when publishing isothermal amplification assays. The frequent failure to report important information and comparable performance data is one of the reasons why further improvement and application of isothermal amplification-based diagnostics is hindered. As these shortcomings represent an obstacle to the adoption of assays, translation into real-world testing and (environmental) applications is exceedingly rare. Even though these amplification techniques were developed as a simpler and low-resource alternative to PCR, their implementation in easy-to-use or lab-on-a-chip systems is still rare.

The aim of our study was not to denigrate or discredit the published assays but to highlight the urgent need for minimum requirements and quality control for publishing isothermal amplification assays to promote and support their applicability in real-world settings. Such troublesome inconsistencies could be avoided by adhering to certain guidelines when publishing newly developed assays, facilitating and enhancing reproducibility and evaluation. In order to promote this approach in qPCR assay development, the MIQE guidelines were published in 2009 (19). As an extension for digital PCR, the digital MIQE guidelines were released in 2013 (43). Even though the first loop-mediated isothermal amplification assay was published in 2000, there are still no corresponding guidelines for isothermal amplification methods. It would be easy and, as demonstrated in our results, recommendable to transfer the existing and well-structured MIQE guidelines to isothermal amplification assays. This would improve comparability and enhance the translation and relevance of research findings. Additionally, guidelines could help end users identify suitable assays for their intended applications and to restore confidence in the utility of isothermal amplification-based diagnostics. As bias and variability are inherent to all measurements, it is necessary to understand the level of error and its impact on the study conclusion, which is not possible when critical information is missing. Therefore, it is crucial to enhance the transparency in reporting, which should be put into practice by researchers, reviewers, and editors. Based on our findings, the minimum information when publishing isothermal amplification should include (i) complete assay composition (reagents, enzymes, primer sequences, and concentrations); (ii) clear definition of sample types and strains used; (iii) DNA input quantity per reaction; (iv) number of replicates; and (v) consistent performance metrics expressed in genome copies or CFU per reaction. There should be a focus on assay validation and cross-comparison under controlled laboratory conditions. This would help to enable future validations to be carried out even with complex matrices in order to establish the originally intended field suitability of isothermal amplification methods. These efforts could lead to closing the gap between technological promise and translational feasibility.

## MATERIALS AND METHODS

### Literature search and assay selection

Before starting the evaluation of different isothermal amplification assays, a qPCR reference method had to be established for benchmarking. After the comparison of different qPCR assays targeting *P. aeruginosa*, the protocol of Lee et al. (34) was chosen. Next, a literature search for published LAMP detecting *P. aeruginosa* was performed in September 2023. Three different databases (Web of Science, PubMed, and Google Scholar) were used to find 22 different candidate assays. As there were more published LAMP assays, LAMP is in much broader use and can be used in a semiquantitative format, we decided to focus on this type of isothermal amplification for *P. aeruginosa* target genes. This narrowed search resulted in the following list of assays: Chen et al., Zhang et al., and Hou et al. (24, 25, 44)*; Zhao et al., Manajit et al., and Wen et al. (26, 27, 45)*; Li et al., Goto et al., Dong et al., Si et al., and Kinoshita et al. (28–31, 46)*; and Zeng et al. (32). It should be noted that three of the assays (marked by *) were excluded as they did not report primer sequences and/or reaction compositions (44–46).

### Bacterial reference strains

Important assay parameters (specificity, sensitivity, and limit of detection) were tested by analyzing the isolated genomic DNA of pure cultures of different bacterial strains. For the first screening step, the different LAMP assays were tested with genomic DNA of *P. aeruginosa* type strain ATCC 10145. The 12 target strains for sensitivity experiments were *P. aeruginosa* environmental isolates 1–6 and *P. aeruginosa* clinical isolates 1–6. The 19 non-target strains for specificity experiments were selected with a focus on the environmental application of the LAMP assays and the reference qPCR method, ranging from bacteria closely related to *P. aeruginosa* to distantly related species, all of which may occur in water or in clinical settings and are therefore relevant for assay application. Therefore, we selected *Escherichia coli* ATCC 11775, *Enterococcus faecalis* ATCC 19433, *Enterococcus faecium* ATCC 19434, *Legionella anisa* ATCC 35292, *Staphylococcus saprophyticus* ATCC 15305, *Klebsiella pneumoniae* ATCC 13883, *Proteus mirabilis* ATCC 29906, *Pseudomonas putida* ATCC 12633, *Pseudomonas fluorescens* ATCC 13525, *Staphylococcus aureus* DSM 20232, *Streptococcus bovis* ATCC 33317, *Citrobacter freundii* ATCC 8090, *Acinetobacter baumannii* ATCC 19606, *Vibrio cholerae* O1 ATCC 39315, *Vibrio mimicus* ATCC 33653, *Vibrio parahaemolyticus* ATCC 17802, *Vibrio vulnificus* ATCC 27562, *Vibrio fluvialis* ATCC 33809, and *Vibrio alignolyticus* ATCC 17749.

### Environmental *P. aeruginosa* isolates

The environmental isolates (*n* = 6) were obtained from water samples taken during water hygiene inspections in healthcare facilities in Lower Austria. *P. aeruginosa* was enumerated according to the ISO 16266 method (47). In brief, 250 mL of water sample was passed through a membrane filter (pore size 0.45 µm, cellulose acetate; Sartorius, Germany) and incubated on selective agar (*Pseudomonas* CN, Oxoid; Thermo Fisher Scientific, UK) for 44 ± 4 h. Presumptive colonies were biochemically examined using the cytochrome-oxidase test (tetramethyl-p-phenylendiamindihydrochloride; Merck, Darmstadt, Germany) and acetamide test (Nessler’s reagent, Merck). Isolates were subcultivated on Columbia Agar (Oxoid, Thermo Fisher Scientific). Afterwards, isolates were heat inactivated (95°C, 5 min), and the cell material was resuspended in 1 mL sterile, ultrapure water.

### Clinical *P. aeruginosa* isolates

Clinical isolates (*n* = 6) were obtained from urine patient samples of the University Hospital St. Pölten (Austria). Bacterial identification was performed using matrix-assisted laser desorption/ionization time-of-flight mass spectrometry (Bruker Daltonics, Germany), according to the manufacturer’s instructions. Isolates were cultured on Columbia Blood Agar Plates (Oxoid, Thermo Fisher Scientific) and incubated at 37°C for 24 h. After incubation, colonies were harvested and resuspended in 1 mL of sterile 0.9% sodium chloride solution for subsequent DNA extraction.

### DNA extraction

The extraction of genomic DNA from pure cultures of the bacterial reference strains as well as from the *P. aeruginosa* isolates was performed by using the QIAamp DNA Mini Kit from QIAGEN (Hilden, Germany) following the manufacturer’s protocol. The isolated DNA was stored at −20°C until further use.

### Quantification of bacterial DNA

After the extraction of genomic DNA strains, bacterial genomic DNA was quantified with a qPCR assay targeting the V1–V2 region of the 16S rRNA gene with primer binding sites universal to all bacteria (33). The qPCR reactions were carried out on a qTOWER³ G real-time thermocycler (Analytik Jena, Jena, Germany) and in a total reaction volume of 15 µL containing each primer at a concentration of 200 nM (Merck) (see Table 2 for oligonucleotide sequences), 7.5 µL KAPA SYBR Fast qPCR Master Mix 2x (Peqlab, Erlangen, Germany), and 2.5 µL sample. The assay was performed according to the following temperature protocol: 3 min at 95°C, followed by 40 cycles of 30 s at 95°C, 30 s at 57°C, 1 min at 72°C, and 2 min at 72°C. Unless otherwise stated, qPCR reactions were carried out in triplicate and no template controls (NTCs) were included in each qPCR run. Although a small number of target copies are detected in each NTC due to *E. coli* DNA residues in the polymerase, these numbers are several orders of magnitude lower than those in the actual samples. Therefore, runs were accepted if the NTCs contained less than 100 copies of the 16S rRNA target per reaction. A dilution series of DNA plasmid solution containing a known number of copies of the 16S rRNA target gene fragment was used to generate a calibration curve. As *P. aeruginosa* has four copies of the 16S rRNA gene, the calibration curve can be consequently used to calculate the genome copies per microliter in the analyzed samples.

### Quantitative PCR reference method targeting *P. aeruginosa*

As a reference method, the qPCR assay of Lee et al. (34) was chosen. The quantitative PCR reactions were carried out in a total reaction volume of 15 µL containing each primer at a concentration of 0.5 µM (Merck), 0.25 µM of the probe (Microsynth, Balgach, Switzerland) (see Table 2 for oligonucleotide sequences), 7.5 µL Luna Universal qPCR Master Mix (New England Biolabs), and 2.5 µL sample. The reactions were performed on a qTOWER³ G Real-Time Thermocycler (Analytik Jena) according to the following temperature protocol: 1 min at 95°C, followed by 44 cycles of 30 s at 95°C and 90 s at 58°C. Unless noted otherwise, qPCR reactions were carried out in triplicate, and NTCs were included in each qPCR run.

### LAMP reaction

All oligonucleotides used in this study are listed in Table 2. After selecting different LAMP assays from the literature search, the compositions of the reactions were extracted. Out of the nine chosen assays, six published all necessary information to reproduce the assay in a reliable manner. Mainly, details regarding the components of the used buffers and the primer concentrations were missing. In general, all reactions were carried out in a total reaction volume of 25 µL, composed of 0.5 µL LAMP dye (New England Biolabs) and 2.5 µL sample DNA. Additionally, 8 U Bst 2.0 DNA polymerase large fragments (New England Biolabs) were used for all assays except one, which published a quantity of 4.8 U. If not reported differently, and also if the buffer composition was not reproducible, 2.5 µL Isothermal Amplification Buffer (New England Biolabs), 1 M betaine (Sigma-Aldrich, St. Louis, USA), 1.6 mM dNTPs (New England Biolabs), and 6 mM MgSO_4_ (New England Biolabs) were added in all reactions. Additionally, for the assay of Manajit et al. (27), no UDG was included, whereas for that of Zeng et al. (32), neither dUTP nor Antarctic Thermolabile uracil DNA glycosylase was included. As Chen et al. (24) did not publish concentrations of any reaction components, we applied the reported volumes and used the following stock solutions: 5 M betaine, 10 mM dNTPs, 100 mM MgSO_4_, 10 µM F3 and B3, as well as 100 µM FIP and BIP. Furthermore, we could not identify which primer concentrations Dong et al. (30) added in the reaction; therefore, we used the reported volumes from 100 µM primer stocks. To ensure a comparison under identical conditions, we decided to apply the same detection method for all assays. A LAMP dye (New England Biolabs) was used to enable real-time fluorescence measurement in a qPCR instrument, as this allowed a simple, sensitive, and reliable comparison and corresponds well with visual assessment of positive reactions.

The LAMP reactions were performed on a qTOWER³ G real-time thermocycler (Analytik Jena). The real-time fluorescence was considered positive if a *C*_*t*_ value could be detected. The automatic manufacturer settings were used to set the thresholds to ensure that there was no bias towards data analysis. Unless noted otherwise, LAMP reactions were carried out in triplicate, and NTCs were included in each run.

### First screening

As a first step of the systematic evaluation, the different LAMP assays were tested with a serial dilution of *P. aeruginosa* ATCC 10145 ranging from 10^6^ to 10^2^ gc/rxn.

### Specificity and sensitivity

To evaluate the assay sensitivity and specificity, the genomic DNA of the selected bacterial reference strains was analyzed with the different LAMP assays and the qPCR reference method using a concentration of 10^3^ gc/rxn. All reactions were carried out in triplicate. The resulting sensitivity and specificity values were calculated using the published mathematical terms by Lemmon and Gardner (35):


$$Sensitivity=1-\text{false-negative rate}=1-\frac{{\text{number of false-negative strains}}^{*}}{\text{number of true-positive strains}\;+\;{\text{number of false-negative strains}}^{*}},$$

$$Sensitivity=1-\text{false-positive rate}=1-\frac{{\text{number of false-negative strains}}^{*}}{\text{number of true-positive strains}\;+\;{\text{number of false-negative strains}}^{*}}.$$

where * indicates that strains are counted as false positive or false negative if at least one replicate is false positive or false negative, respectively.

### LOD experiments

The limit of detection was determined for the different LAMP assays and the qPCR reference method. Hereby, dilutions of genomic DNA with known concentrations from three different *P. aeruginosa* strains (ATCC 10145, clinical isolate 1, and environmental isolate 1) were used, whereby each dilution was measured in 12 replicates. For the qPCR reference method, ATCC 10145 was analyzed with twofold dilutions from 39.6 to 0.31, clinical strain 1 from 60 to 0.47 and environmental strain 1 from 43.75 to 0.34 gc/rxn. The limit of detection of the LAMP assay from Manajit et al. (27) was determined with twofold dilutions of the ATCC 10145 ranging from 5.76 × 10^4^ to 7.03, clinical strain 1 from 9.60 × 10^2^ to 1.88, and environmental strain 1 from 2.80 × 10^3^ to 2.73 gc/rxn. As for the LAMP assay from Dong et al. (30), ATCC 10145 was measured in twofold dilutions from 7.20 × 10^3^ to 7.03, clinical strain 1 from 9.60 × 10^2^ to 0.94, and environmental strain 1 from 1.40 × 10^3^ to 0.68 gc/rxn. For the determination of the threshold where 95% of the replicates delivered a positive signal (LOD_95_), a logistic regression model was used (48).

### Statistical analysis

Calculations and tables were accomplished in Microsoft Excel. Limit of detection calculations were performed in R 4.4.1 (49). Additionally, the graphical abstract was generated with BioRender. qPCR data were analyzed with qPCRsoft 4.0.

## Data Availability

The raw data of the analyzed LAMP assays can be found on the TU Wien Research Data repository under DOI https://doi.org/10.48436/2qqyd-pc940.

## SUPPLEMENTAL MATERIAL

Supplemental material spectrum.03235-25-s0001.docx Table S1 and Fig. S1.

ASM does not own the copyrights to Supplemental Material that may be linked to, or accessed through, an article. The authors have granted ASM a non-exclusive, world-wide license to publish the Supplemental Material files. Please contact the corresponding author directly for reuse.
