# Supplementary material for: Reproducibility crisis in isothermal amplification: lessons from benchmarking LAMP assays
Source: Microbiol Spectr. 2026 Apr 27;14(6):e03235-25. doi: 10.1128/spectrum.03235-25 (PMC13228004; doi:10.1128/spectrum.03235-25)
Supplement: Supplemental material — Table S1 and Fig. S1. [file spectrum.03235-25-s0001.docx]

**Supplementary Information**

**Reproducibility crisis in isothermal amplification:**

**Lessons from benchmarking LAMP assays**

Lena Piglmann^a,h^, Lena Campostrini^c,h^, Regina Sommer^d,h^ Alexander Kirschner^c,e,h^, Rudolf Krska^f,g^, Andreas H. Farnleitner^b,e,h^, Claudia Kolm^e,h^, Georg H. Reischer^a,h^

^a^ TU Wien, Institute for Chemical, Environmental and Bioscience Engineering 166-05-3, Working Area Molecular Diagnostics, IFA Tulln, Tulln, Austria,

^b^ TU Wien, Institute for Chemical, Environmental and Bioscience Engineering, Research Group Microbiology and Molecular Diagnostic 166/5/3, Vienna, Austria,

^c^ Medical University of Vienna, Centre for Pathophysiology, Infectiology and Immunology, Institute for Hygiene and Applied Immunology - Water Microbiology, Vienna, Austria,

^d^ Medical University of Vienna, Centre for Pathophysiology, Infectiology and Immunology, Institute for Hygiene and Applied Immunology - Water Hygiene, Vienna, Austria,

^e^ Karl Landsteiner University of Health Sciences, Division Water Quality and Health, Krems, Austria,

^f^ University of Natural Resources and Life Sciences Vienna (BOKU), Institute of Bioanalytics and Agro-Metabolomics, Department of Agricultural Sciences, IFA Tulln, Tulln, Austria,

^g^Institute for Global Food Security, School of Biological Sciences, Queens University Belfast, Belfast,
Northern Ireland BT7 1NN, UK,

^h^ICC Interuniversity Cooperation Centre Water & Health, Vienna, Austria, https://www.waterandhealth.at/.

Corresponding author:

Georg H. Reischer

georg.reischer@tuwien.ac.at

Contents

**Table S1** Reaction compositions of the different LAMP assays targeting P. aeruginosa, listed are all published components of the LAMP reactions as they are stated in the cited publications; the units of the components have been unified as good as possible to enable a better comparison.

**Figure S1a-c** Graph showing the results for qPCR obtained from the analysis of the *P. aeruginosa* gDNA dilution series

Table S1: Reaction compositions of the different LAMP assays targeting P. aeruginosa, listed are all published components of the LAMP reactions as they are stated in the cited publications; the units of the components have been unified as good as possible to enable a better comparison. The indication N/A means, that no information was available for this section.

|  | **Chen et al.**  **(24)** | **Zhang et al.**  **(25)** | **Zhao et al.**  **(26)** | **Manajit et al.**  **(27)** | **Li et al.**  **(28)** | **Goto et al.**  **(30)** | **Dong et al.**  **(31)** | **Si et al.**  **(32)** | **Zeng et al.**  **(33)** |
| --- | --- | --- | --- | --- | --- | --- | --- | --- | --- |
| Water | 0.5 µL |  |  |  |  |  | 9.4 µL |  | 8.5 µL |
| Betain | 3 µL | 1 M | 1 M | 0.8 M |  | 0.8 M |  |  |  |
| dNTPs | 2.3 µL | 1.6 mM | 1.6 mM | 1.4 mM | 1.4 mM | 1.4 mM |  |  | 5.6 mM |
| dUTP |  |  |  |  |  |  |  |  | 1.4 mM |
| Thermopol Buffer (NEB) | 1.5 µL | 2.5 µL | 1 x | 1 x | 2.5 µL |  |  | 2.5 µL | 2.5 µL |
| Chromogenic reagent |  | 2 µL |  |  |  |  |  |  |  |
| Mixture (with ddH_2_0, Mg^2+^) |  |  |  |  |  |  |  | 18.5 µL |  |
| IsoAmp buffer (TransGen Biotech) |  |  |  |  |  |  | 12.5 µL |  |  |
| SYBR Green |  |  | Yes, but N/A |  | Yes, but N/A |  |  | 1 µL |  |
| Evagreen dye (20x) |  |  |  |  |  |  |  |  | 1.25 µL |
| Tris HCl |  |  |  |  |  | 20 mM |  |  |  |
| KCl |  |  |  |  |  | 10 mM |  |  |  |
| (NH_4_)_2_SO_4_ |  |  |  |  |  | 10 mM |  |  |  |
| Tween 20 |  |  |  |  |  | 0.1% |  |  |  |
| MgSO_4_ | 0.3 µL | 6 mM | 6 mM | 5 mM | 5.2 mM | 8 mM |  | 8 mM | 6 mM |
| Monomeric Cyanine |  |  |  |  |  |  |  |  |  |
| Hyroxy naphtol blue trisodium salt |  |  |  |  |  | 120 µM |  |  |  |
| Antartic Thermolabile uracil DNA glycosylase |  |  |  |  |  |  |  |  | 0.5 µL of 1 U/µL |
| UDG |  |  |  | 5 U |  |  |  |  |  |
| Bst Pol | 0.6 µL | 8 U | 8 U | 8 U | 8 U | 8 U | 8 U | 8 U | 8 U |
| F3 / B3 | 0.3 µL | 0.2 µM | 0.2 µM | 5 pmol | 0.2 µM | 0.2 µM | 0.1 µL | 0.2 µM | 0.2 µM |
| FIP / BIP | 2.3 µL | 1.6 µM | 1.6 µM | 40 pmol | 1.6 µM | 1.6 µM | 0.2 µL | 1.6 µM | 1.6 µM |
| LF / LB |  |  | 0.8 µM |  |  | 0.8 µM | 0.1 µL |  | 0.8 µM |
| DNA Sample | 1.2 µL | 2 µL | 1 µL | 1 µL | N/A | 1 µL | 1 µL | 1 µL | 2 µL |


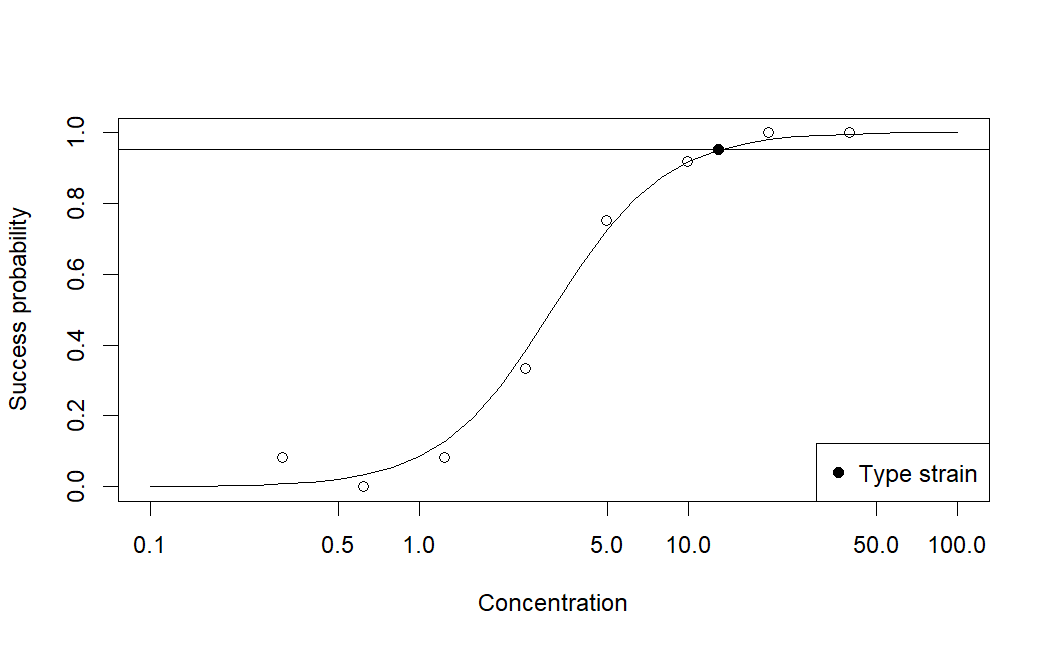


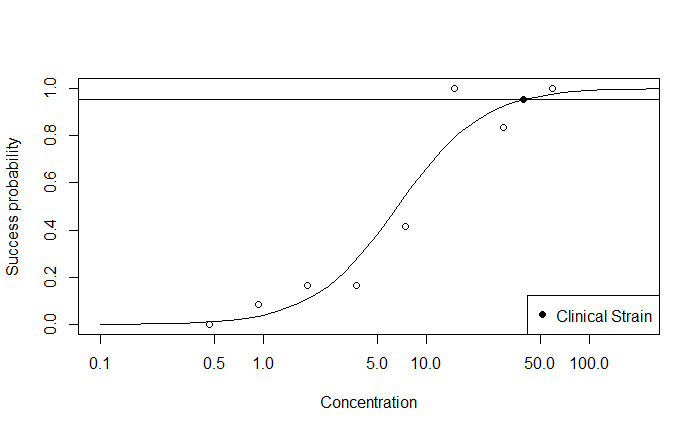


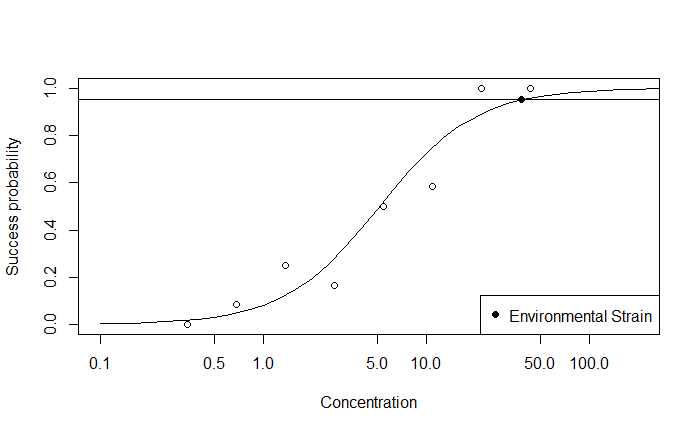


Figure S1a-c: Graph showing the results for qPCR obtained from the analysis of the P. aeruginosa gDNA dilution series. The horizontal line with the filled symbol indicates the limit of detection where 95% of the replicates are positive (LOD_95_). For this statistical determination, a logistic regression model was used.
